# Augmented reality-based software (Echo-QR) for guiding the echographic probe toward the acoustic window: a pilot study

**DOI:** 10.3389/fmedt.2024.1287851

**Published:** 2024-07-05

**Authors:** A. Thevenon, F. Derache, O. Faucoz, K. Zuj, D. Chaput, P. Arbeille

**Affiliations:** ^1^ MEDES-IMPS—Bâtiment Waypost, Toulouse, France; ^2^ CADMOS-CNES—18 Av E Belin, Toulouse, France; ^3^ UMPS-CERCOM Faculté de Medecine, Université de Tours, Tours, France

**Keywords:** echography, organ, acoustic windows, augmented reality, novice, autonomy

## Abstract

**Introduction:**

With current technology, ultrasound imaging in remote conditions, such as the International Space Station, is performed with vocal guidance or using a teleoperated echograph controlled by an expert. Both methods require real-time communications between the novice operator and expert to locate the probe over the appropriate acoustic windows (AW). The current study presents the development and testing of a new augmented reality software (Echo-QR) that would allow novice operators (with no medical imaging background) to correctly locate the ultrasound probe over the AW of interest without expert assistance.

**Methods:**

On the first day of the study, the positions of the probe over the AWs were identified for each organ by an expert sonographer and saved in the Echo-QR software. On the second day, the novices independently performed the ultrasound investigation using the Echo-QR software to correctly position the probe over each organ’s AW.

**Results:**

Using the Echo-QR software, novice operators found the AW in 73 (92%) of the 79 organs. The 2D images acquired by the novices “2D direct image” were acceptable for medical evaluation in 41% of the cases. However, when the “2D direct image” did not show the entire organ, a 3D capture of the volume below the probe was also performed, which allowed for the extraction of the appropriate 2D image “2D/3D image” for medical evaluation in 85% of the cases.

**Discussion:**

Therefore, in the absence of real-time communication between an isolated participant and an expert sonographer, novel software (Echo-QR) and automated 3D volume capture can be used to obtain images usable for ultrasound diagnostics.

## Introduction

With an increase in the duration of spaceflight missions to the International Space Station (ISS), and the future missions to the Moon and Mars, ultrasound imaging will be frequently used for medical monitoring and diagnostics of astronauts. With current technology, reliable investigations of most of the superficial organs (carotid, intracranial, and leg arteries; skeletal muscles) and the deep organs (liver, aorta, kidney, spleen, pancreas, and pelvis) can be performed using three methods: remote guidance, tele-echography, and 3D volume capture. For remote guidance, a trained sonographer verbally instructs the astronaut through the examination on how to locate the probe on the acoustic window (AW), get the right orientation, and activate the ultrasound settings and functions (1). During tele-echography, the trained sonographer on the ground provides verbal instruction to the astronaut for probe placement on the AW only and then controls the motorized probe orientation as well as the ultrasound settings and functions (2). For the 3D volume capture, the astronaut again locates the probe on the AW under the vocal guidance of a trained sonographer on the ground and then activates a scan function where a video of the area under the probe is recorded for later 3D reconstruction and analysis (3).

Before starting any of the three currently available methods of remote ultrasound imaging in space, the novice operator (who has no medical or imaging background) is first required to locate the ultrasound probe over the appropriate AW. In all three methods, this is accomplished with vocal instruction from the ground expert using basic anatomical landmarks (mammillary, axillary, and xiphoid lines, costal border, belly button, etc.) and verbal corrections (1–3). However, even with real-time verbal assistance, finding the AW can be challenging and take several minutes, potentially extending the duration of the examination beyond the time available, as once the probe is located correctly, adjustments are still required to obtain images for evaluation. This can become even more challenging if a communication lag exists between the expert on the ground and the novice operator.

Various methods have been proposed to assist novice operators when acquiring images, for example with techniques that can be used when real-time communication with an expert center is not available. In one method, images obtained by the novice operator are compared to ideal images stored on the ultrasound system, with the system indicating if the acquired image is acceptable based on this comparison (4). Another group has worked to develop a teleoperated ultrasound system that uses augmented reality to project a virtual probe (whose location and orientation have been previously selected by an expert) to assist the novice operator in finding the proper probe location and orientation (5). Other work has used CT images that are virtually projected onto the participant’s body, allowing for direct visualization of organ locations (6); however, the need for original CT scans can be constraining and counterproductive to the original purpose of ultrasound scanning. Some groups have used other methods based on 3D technology to visualize the desired and actual probe orientation (7). Ford et al. (8) also worked on a similar concept with HeartPad: a dynamically deformed 3D mesh of the heart based on real-time anatomical feature identification that can help general practitioners or untrained users correlate anatomy with ultrasound images. Unfortunately, all of the methods listed above required the real-time assistance of a trained sonographer to initially locate the ultrasound probe over the AW.

The objective of the present study was to assess the ability of a novel software, “Echo-QR,” developed by our group to assist an untrained operator in locating the probe on the AW of interest on his or her own. It was hypothesized that the camera-based tracking system and a dedicated human–machine interface (HMI) would be sufficient for the novice operator to independently reproduce both the probe location and orientation previously recorded by an expert sonographer.

## Research design and methods

### Participants

A total of 10 participants were included in the study (8 men, 2 women; mean age 34 ± 7 years, mean height 170 ± 10 cm, mean weight 70 ± 15 kg). Eight different organs were examined in each participant; thus, the Echo-QR software was tested on 79 different organs. All were informed about the experimental procedures and gave their written informed consent. The experimental protocol conformed to the standards set by the Declaration of Helsinki and was approved by the local Ethics Committee (2021-060—University of Tours France).

### The Echo-QR software

The software, Echo-QR, is based on a camera-tracking development kit, Vuforia (PTC, Boston, MA, USA). The Echo-QR software was developed in-house using the Unity game development tool as well as the Vuforia augmented reality development kit. The coding language used is C#. Vuforia was used to efficiently detect and track the QR cubes. The rest of the Echo-QR interface was coded by the team. Echo-QR has been installed on a Samsung Z Fold 2 5G. The camera used is the built-in camera of the Samsung smartphone.

**Figure 1:**
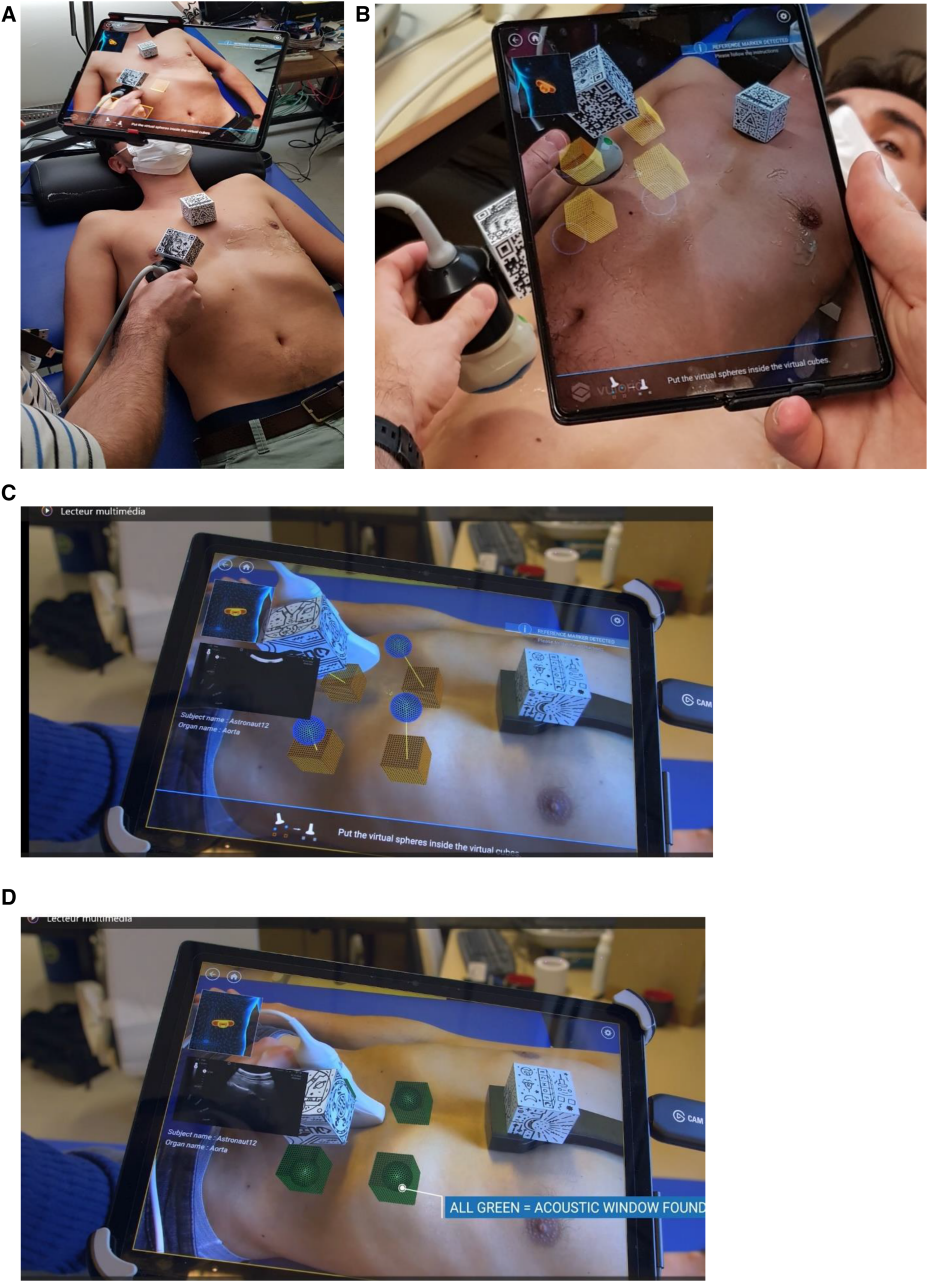
(**A**,**B**) View of the setup: QR cube fixed on the ultrasound probe body and on the chest support, and the Echo-QR software running on a smartphone/tablet placed on an articulated arm. During the acquisition phase, an expert saves probe positioning data. The relative position of the two QR cubes is displayed, corresponding to the four cubes on the tablet screen. During the restitution phase (**C**,**D**), virtual cubes indicate the probe positioning previously saved by the expert sonographer, and spheres indicate the current position of the probe. The novice operator moves the probe until the four spheres align with the four corresponding cubes and turn green, indicating proper alignment and orientation (**C,D**). The echographic image of the organ appears on the smartphone/tablet.

This system allows for the detection and tracking of the position and orientation of specific multi-targets called QR cubes. With one QR cube placed on the probe (probe QR cube) and another one on the chest of a participant (reference QR cube) (Figure 1A), the probe location and orientation are determined by the relative positions of the two QR cubes and can be recorded using the Echo-QR software installed on the smartphone/tablet. Using Echo-QR software requires two phases: acquisition (Figure 1B) and restitution (Figure 1C). During the acquisition phase, an expert sonographer locates the ultrasound probe over the AW of the organ to be investigated and changes the orientation of the probe until the perfect 2D view of the organ for medical assessment appears on the screen (2D direct view). The software then determines the location and orientation of the probe QR cube in relation to the reference QR cube and saves these data as the perfect position and orientation of the probe. During the restitution phase, the novice operator uses the previously saved data to replicate the probe position and orientation.

To replicate the positioning of the probe, the novice operator uses a dedicated human–machine interface. The interface includes the following: (1) a contextual view (top-left of the screen) with an approximate probe positioning on a 3D human body for the investigated organ; (2) notifications (top right of the screen) to let the novice know the tracking status of both QR cubes; (3) instructions (bottom of the screen) to guide the novice to the next action to perform; and (4) an augmented reality view (center of the screen) with the desired probe position represented by four cubes and the actual probe position represented by four spheres.

**Figure 2:**
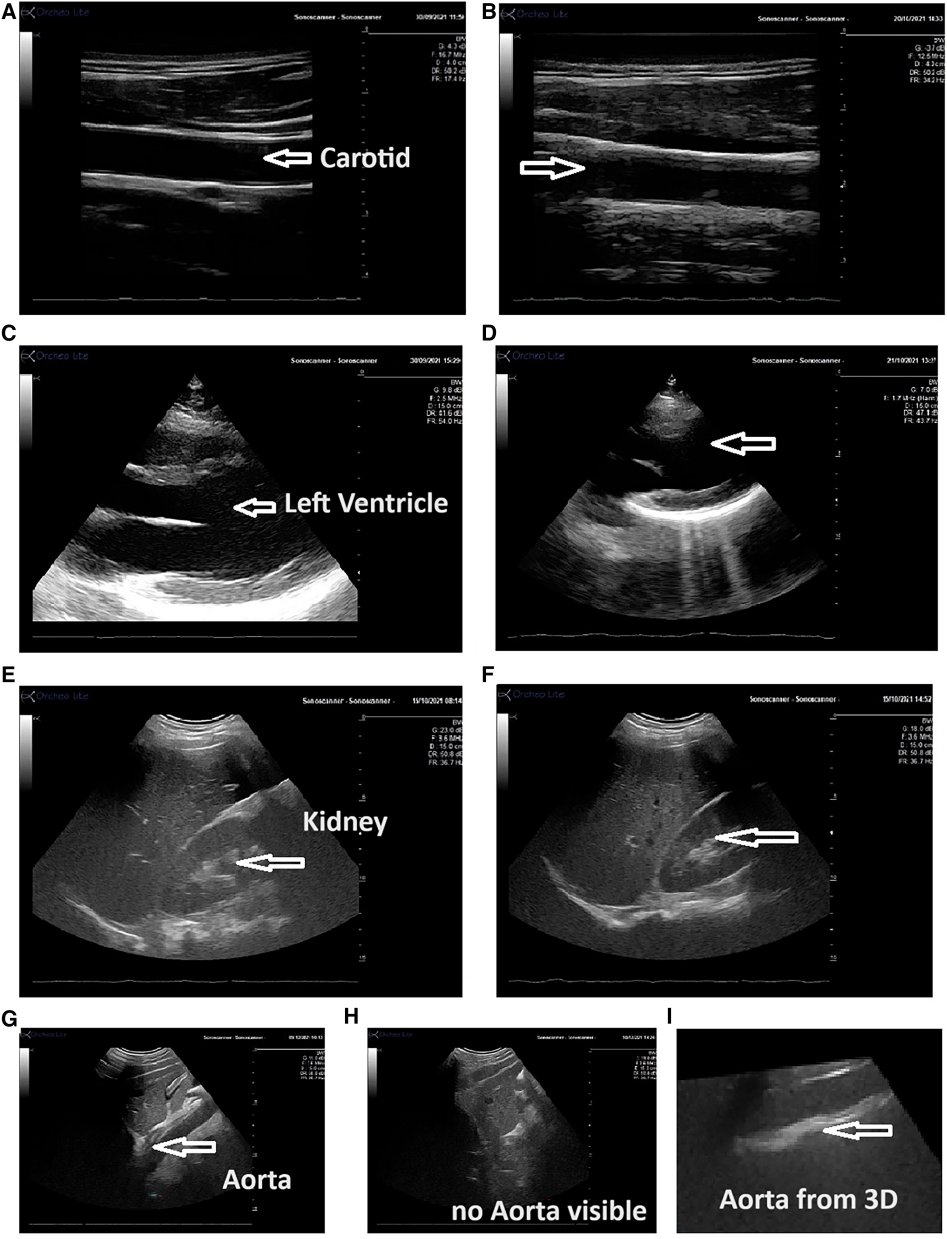
Echographic view obtained by the expert and the novice operator using the Echo-QR application. The images show the carotid artery [(**A**) expert; (**B**) novice], the left ventricle [(**C**) expert; (**D**) novice], kidney [(**E**) expert; (**F**) novice], and the abdominal aorta [(**G**) long-axis view obtained by the expert, (**H**) novice using the Echo-QR only obtained an image close to the aorta area but the image of the aorta was not displayed, and (**I**) the 3D capture at this location provided the desired long-axis view of the aorta]. In all cases, the quality of the vessel or organ image (resolution contrast) was similar for both the expert and novice operator.

**Figure 3:**
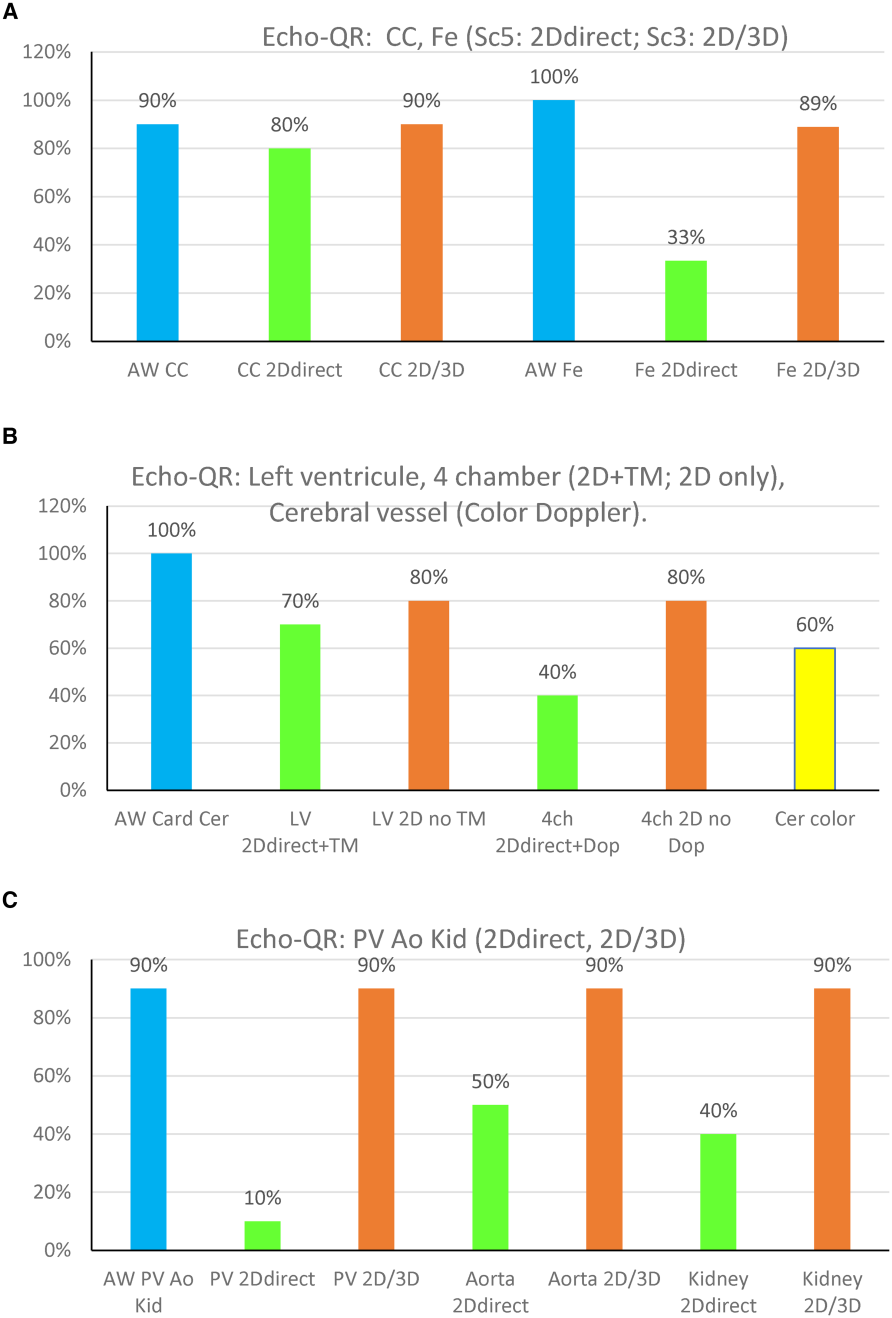
Echo-QR findings organ by organ on the 10 participants. (**A**) CC, common carotid; Fe, femoral artery. (**B**) LV, left ventricle long-axis, and 4 Ch, four-chamber; Cer color, cerebral view in color Doppler. (**C**) PV, portal vein, aorta; Kid, kidney. “2D direct”: 2D image acquired directly by the novice while using the Echo-QR soft. “2D/3D”: 2D image extracted from the 3D capture. The % on the OY axis is ratio between the number of good images/number of images for each organ.

To correctly position the probe, the novice operator moves the probe (up/down/right/left) until each sphere aligns with its corresponding cube (Figures 1C,D). At this point, the probe position and orientation closely match the ones saved by the expert in the initial acquisition phase. When a sphere aligns with the appropriate cube, they both turn green and when all spheres are aligned, the ultrasound image taken during the acquisition phase is shown on the screen (2D direct view) for comparison (Figure 1D). If the image acquired by the novice operators does not match the 2D direct view, the novice triggers a 3D capture of the volume below the probe. During post-processing, the expert sonographer then analyzes the 3D volume reconstruction to obtain the correct 2D view for assessment of the “2D/3D image” (Figures 2, 3).

The Echo-QR software can run on any device supported by Vuforia, but the tracking quality of the QR cubes is directly related to the video quality of the camera. In addition, as the probe orientation is determined with respect to the reference cube, a support structure was designed to consistently (within 10 mm) locate the cube on the participant's sternum between the xiphoid process and the manubrium.

### Experimental protocol

The experiment took place over 2 days. On the first day, Echo-QR acquisition data were collected by a sonographer on the novice participants after 20 min of resting supine. For acquisition, the reference QR cube was fixed to the participant’s sternum using the support structure. The Echo-QR software was run on a smartphone (Samsung Z Fold 2 5G, Samsung, Suwon-si, Gyeonggi-do, Korea) supported by an articulated arm (Articulated phone holder, Guangfanouzhou, Hangzhou, Zhejiangshen, China). During this session, the expert sonographer located the probe on the AWs for eight different organs (on each of the eight participants) and displayed a perfect 2D view of each [carotid and femoral arteries, long-axis and four-chamber views of the heart, transcranial Doppler (TCD), portal vein, aorta, and kidney]. The probe positioning and orientation (represented by the relative position of the probe and reference QR cubes) for each organ were recorded using the Echo-QR software. On the second day, participants performed the ultrasound investigation independently using the Echo-QR software to locate and orient the probe on each organ (Figure 2). The novice was trained to use the software (they were taught how to put the virtual spheres inside the virtual cubes) but on fake targets. The novice was not trained to target specific organs.

### Image evaluation

Each image acquired by the novice operator was evaluated as to whether the AW was found correctly, the quality of the “2D direct image” or 2D image extracted from the 3D reconstruction “2D/3D image,” and the quality of the image compared to the image obtained by the expert sonographer. For the AW assessment, images were scored as 5 if the AW was found (part of the organ visible) or 0 if the AW was not found (no organ image at all). Compared to the expert image, the novice image was scored as 5 if the view was similar to that of the expert, 3 if the view was of lower quality (incomplete view, lower contrast but medically usable), or 1 if it was not usable for medical diagnosis.

For superficial neck targets and for deep organs, the quality of the images acquired by the novice operator was scored as 5 if there was a 2D direct perfect organ view (similar to the expert one) that could be used immediately for Doppler or time-motion display. Images were scored as 3 if there was a partial organ view that allowed for the 3D capture to produce an appropriate 2D view usable for medical diagnosis. Finally, the image was given a score of 1 if the 2D and 3D capture images were not usable for medical diagnosis. For the left ventricle long-axis and four-chamber views of the heart, images were scored 5 when the image allowed a time-motion or Doppler recording, or 3 if the cardiac structure was visible but the image was not acceptable for time-motion capture.

## Results

The Echo-QR software was used to assess eight different organs; however, one structure in one participant was not imaged due to poor echogenicity. Therefore, only 79 of the expected 80 organs (10 participants × 8 organs) were investigated by both the expert sonographer and the novice operator. Examples of the ultrasound images obtained by the expert sonographer and the novice operator are presented in Figure 2.

The Echo-QR application allowed the novice user to find the acoustic window for 73 (92%) of the 79 scans. The percentage of images scoring 5 (2D direct image with a full view of the organ, with possible Doppler) and 3 (2D image extracted from 3D capture “2D/3D image”) for each of the eight organs is shown in Figure 3.

**Figure 4:**
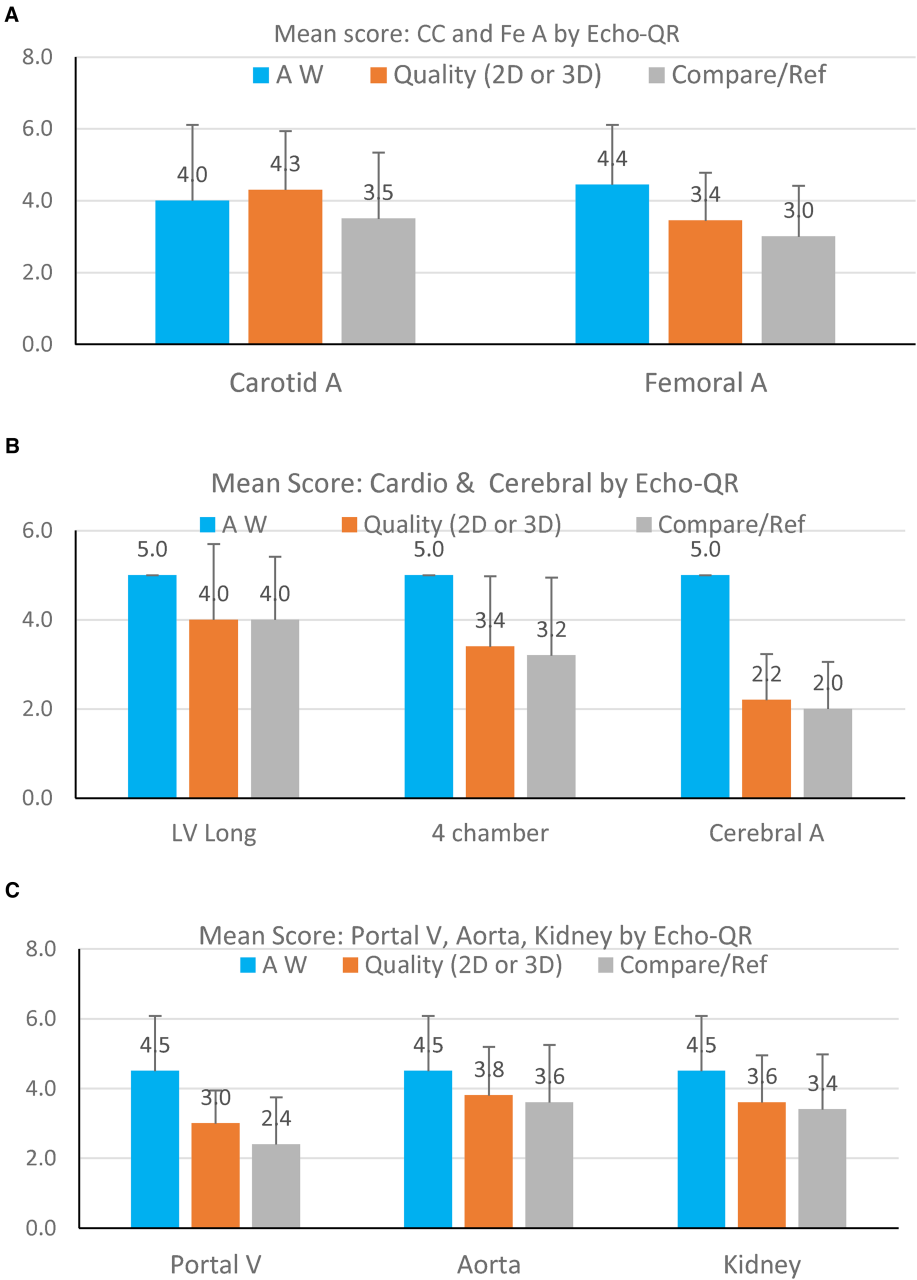
Mean image scores for finding the AW (blue bars), quality of the 2D image (orange bars), and quality of the image as compared to the expert sonographer (gray bars). Data are presented for each organ visualized: (**A**) carotid artery and femoral artery; (**B**) cardiac long-axis, cardiac four-chamber view, and cerebral artery; (**C**) portal vein, abdominal aorta, and kidney. The score on the OY axis ranges from 1–5.

The 2D direct images acquired by the novice operator were of sufficient quality for Doppler or time-motion analysis in 32 (41%) of the 79 images. The organ views obtained directly by the novice or extracted from the 3D capture were found to be acceptable for medical use (score 3) in 67 (85%) of the 79 cases. At the same time, images were generally of lower quality than those obtained by the expert sonographer (Figure 4), but they allowed for medical diagnosis. Only 12 (15%) images were of insufficient quality for medical use.

### Common carotid artery

Echo-QR found the acoustic window in 90% of the participants. A perfect 2D view (score 5 = 2D direct with possible Doppler) was found for 80% of the scans. Echo-QR found an acceptable 2D view (score 5 or 3 = 2D direct or 2D via 3D) in 80% of the scans.

### Femoral artery

Echo-QR found the acoustic window in all participants (100%). Perfect 2D views (score 5 = 2D direct with possible Doppler) were found for 30% of the scans, with acceptable views (score 5 or 3, 2D direct or 2D via 3D) for 80% of the scans.

### Heart imaging

The acoustic windows for the left ventricle long-axis view and the four-chamber view were found in all scans (100%). A perfect 2D view (score 5 = 2D direct and time-motion) for the long-axis view was found in 70% of the scans and 40% of the scans for the four-chamber view (score 5 = 2D direct and possible Doppler). Acceptable views (score 3 = 2D view only, without Time Motion or Doppler) were found in 80% of the long-axis scans and 80% of the four-chamber scans.

### Cerebral arteries

Echo-QR found acceptable 2D views with possible color Doppler in 60% of the scans.

### Abdominal imaging

Abdominal imaging involved assessments of the portal vein, aorta, and kidney. The appropriate AW was found in 100% of the scans for all three target organs. Perfect 2D direct images were not acquired for the portal vein but were found in 50% of the aorta scans and 40% of the kidney scans. Acceptable images (2D direct or 2D/3D image) were acquired in 90% of the portal vein, aorta, and kidney scans.

## Discussion

The purpose of the current study was to evaluate the ability of novel software to assist novice ultrasound operators in independently finding acoustic windows and acquiring ultrasound images for medical assessment. Consistent with the hypothesis, the use of Echo-QR allowed the novice operators to find the AWs in 92% of the cases. This was the first and main objective of the project, as it provided a solution to the weakness (finding the AW) of all the other methods used to perform remote ultrasound investigations.

Positioning of the probe using Echo-QR resulted in perfect 2D images for analysis in 41% of cases. However, the addition of an automated scan increased the percentage of medically usable 2D images (2D direct or 2D/3D) to 85%. These results indicate that the novel Echo-QR software is a viable method for providing automated assistance to novice ultrasound operators in correctly positioning the probe and obtaining images for medical assessment.

Acquiring ultrasound images for medical diagnosis requires both the proper placement of the probe on the patient's body and then fine adjustments (mainly orientation) to optimize the image for assessment. Due to their portability, many modern ultrasound systems are ideal for medical assessments in remote environments. As these isolated environments frequently do not have access to expert sonographers to perform the assessments on site (e.g., spacecraft, isolated polar or desert sites, Amazonian areas, and rural locations), methods have been developed to assist novice operators in performing ultrasound investigations. Both remote guidance (1) and teleoperation (2) have been used successfully for ultrasound examinations on the International Space Station but require the novice operator to first locate the probe on their body over the appropriate acoustic window. To assist astronauts in locating the probe, an anatomical map, developed from scans of 300 patients, was used, which indicates the general location of the AW within a diameter area of 5–6 cm (3). However, remote ultrasound investigations generally still require real-time assistance by an expert to acquire images of sufficient quality for medical assessment.

Several programs have been created to assist novice operators in correctly positioning the ultrasound probe. The “AI Caption” software (4) uses image recognition to notify the novice operator when the image they are acquiring is acceptable based on recorded images taken by an expert sonographer. Another software uses machine learning and real-time ultrasound image segmentation (9) to verify the images obtained by the novice operator. While these methods verify the images obtained, they do not guide the novice operator toward the AW or correct the orientation of the probe. In contrast, the Echo-QR software presented in this paper provides feedback to the novice operator regarding the positioning and orientation of the probe and provides guidance for finding the AW of interest and acquiring the desired image.

Using the Echo-QR application, operators see the two QR cubes (reference and probe QR cubes) on the mobile device screen and the real probe that they are manipulating. With this perspective, it becomes easier for the novice operator to align the ultrasound probe representation (spheres) with the four targets (cubes), corresponding to the position previously found by the expert. Once alignment is complete, the novice operator has successfully positioned the probe and oriented it to provide a long- or short-axis view of the organ. Then, if the 2D image is good, the participant can trigger additional functions for analysis, such as pulsed Doppler or a time-motion.

Using the Echo-QR software resulted in perfect views of the target organ in less than half of the scans. Despite this performance being lower than expected, locating the probe on the acoustic window with Echo-QR did allow the user to trigger a volume scan of the area below the probe. This scan was used to create a 3D reconstruction that could then be analyzed by a trained sonographer. By moving a virtual plan inside this reconstructed volume, the trained sonographer could display 2D images of the organ and acquire the appropriate image for medical diagnostics (3). Although the 3D processing does not allow for Doppler or time-motion assessments, it did produce 2D images of sufficient quality for medical evaluations in 85% of the images.

Although assessments in the current study were only performed on a limited number of organs (*n* = 79), the use of the Echo-QR application resulted in a high success rate in finding the AW and providing images for analysis.

There was an overall reduction in the quality of images obtained by the novice operator, dependent on the structure investigated. The highest image quality scores were found for the superficial vessels (carotid and femoral arteries), the abdominal aorta, and the kidney. Higher-quality images for the carotid and femoral arteries were expected, as the superficial vessels are generally easier to visualize for novice operators. It is surprising that the abdominal aorta and kidney images were of high quality, which indicates the effectiveness of using the Echo-QR application to locate these deep structures. In contrast, images of the portal vein and cardiac structures only scored slightly above 3, indicating that these organs are more difficult to visualize.

The average quality score for the cerebral vessels was below 2, indicating that, even with the Echo-QR application, the novice operators had great difficulties finding the appropriate position for the probe. In contrast to other acoustic windows, the temporal bone window for the cerebral vessels is narrow and requires a more accurate orientation of the probe to acquire images. In addition to the AW for cerebral vessels being very small, minor differences between the position of the expert and the novice probe locations can greatly affect the resulting images. In the future, an additional reference QR cube located on the head may be required for imaging the cerebral vessels.

The Echo-QR application presented in this study, based on augmented reality, allows a novice operator to independently locate an ultrasound probe over an acoustic window and get images for medical diagnosis directly or through 3D capture and reconstruction. This contrasts with other applications that use augmented reality but still require real-time communication with an expert sonographer (5–8, 10). Therefore, when real-time communication is not possible, the Echo-QR software can be used to obtain images for medical diagnosis.

## Limitations

The Echo-QR software is dependent on a consistent relationship between the reference cube placed on the participant’s body and the acoustic window. While the reference cube is fixed to a support on the participant's sternum, it is not perfectly stable due to respiration and potential changes in body position. Therefore, changes in the reference cube location with respect to the acoustic window may account for the AW not resulting in images for diagnosis in 15% of scans.

## Conclusion

The current study demonstrated the ability of novel software to assist novice ultrasound operators in acquiring images for medical diagnosis on their own. The Echo-QR software allowed novice ultrasound operators to locate various acoustic windows and successfully acquire images for analysis. As the portability of modern ultrasound systems makes them ideal for use in remote locations, the Echo-QR software will help operators obtain images without any assistance. Therefore, the use of this software will allow for reliable ultrasound medical surveillance during space exploration, where real-time connections to expert sonographers are not available.

## Data Availability

The raw data supporting the conclusions of this article will be made available by the authors, without undue reservation.
